# Comparative Analysis of Pulmonary Function in Rice Mill Workers Versus Unexposed Controls: A Pilot Cross-Sectional Study

**DOI:** 10.7759/cureus.105901

**Published:** 2026-03-26

**Authors:** Prasath Sekar, B Jaya, Sumitra Sudharkodhy, Nishanth Murugan

**Affiliations:** 1 Physiology, Karpaga Vinayaga Institute of Medical Sciences and Research Centre, Madhuranthagam, IND

**Keywords:** milling, occupational exposure, pulmonary function, respiratory health, restrictive lung disease, rice mill workers, spirometry

## Abstract

Introduction

Rice milling is a major agro-based industry in India and employs a large number of workers who are chronically exposed to organic and inorganic dust generated during milling operations. Inhalation of rice husk dust, silica residues, fungal spores, and other airborne contaminants may contribute to airway inflammation, fibrosis, and ventilatory impairment. However, standardized spirometric data from Indian rice mill workers remain limited. This study aimed to compare pulmonary function parameters between rice mill workers and unexposed controls.

Materials and methods

A cross-sectional study was conducted at the Department of Physiology, Karpaga Vinayaga Institute of Medical Sciences, Madhuranthagam. A total of 100 male participants aged 30-60 years were included; of these, 50 were exposed, and 50 were unexposed. Exclusion criteria included smoking, pre-existing respiratory or cardiac disease, inability to perform spirometry adequately, prior exposure to other occupational dust, and recent or active acute respiratory infection. Spirometry was performed using a Medicaid Smart Soft USB software spirometer (Medicaid Systems, Jalandhar, Punjab, India), measuring forced vital capacity (FVC), forced expiratory volume in one second (FEV₁), FEV₁/FVC ratio, and peak expiratory flow rate (PEFR) for both groups. Data were analyzed using SPSS Version 31 (IBM Corp., Armonk, NY, US).

Results

The PFT of the Rice mill workers showed a significant decrease in FVC (1.96 ± 0.22 L), FEV₁ (1.69 ± 0.21 L), and PEFR (4.47 ± 1.89 L/s) compared to controls (*p* < 0.05). However, FEV₁/FVC ratio is more than 70%, suggesting a restrictive pattern of lung involvement, with a restrictive spirometric pattern.

## Introduction

Occupational lung diseases continue to represent a substantial public health burden in developing countries, where rapid industrialization frequently outpaces the implementation of effective workplace safety measures. In such settings, inadequate engineering controls, poor ventilation, and limited regulatory enforcement contribute to sustained exposure to airborne contaminants. Among vulnerable occupational groups, rice mill workers constitute a large yet under-recognized workforce chronically exposed to respirable dust across multiple stages of processing. Rice milling involves sequential operations, including cleaning, drying, hulling, polishing, and packaging, each generating considerable quantities of airborne particulate matter. The resulting dust is a complex mixture containing cellulose particles, husk fibers, crystalline silica, microbial components, and endotoxins capable of inducing acute airway irritation as well as chronic inflammatory and fibrotic lung injury [1,2].

In India, rice milling is predominantly conducted in semi-urban and rural regions where occupational health infrastructure remains limited. Small- and medium-scale mills often lack adequate dust extraction systems or enclosed processing units, resulting in continuous accumulation of particulate matter within the work environment. Concentrations of fine (PM2.5) and coarse (PM10) particles in such settings frequently exceed recommended occupational exposure thresholds established by regulatory authorities. Sustained inhalation of respirable particles promotes airway inflammation, epithelial injury, and progressive remodeling of lung parenchyma, potentially culminating in fibrosis and irreversible reductions in lung compliance. Epidemiological investigations have documented a higher prevalence of chronic respiratory symptoms, such as persistent cough, breathlessness, and chest tightness, along with spirometric patterns suggestive of restrictive impairment among rice mill workers [3,4].

The pathogenesis of pulmonary dysfunction in this population is multifactorial. Mechanical irritation from inhaled dust particles provokes chronic airway inflammation, while microbial contaminants and endotoxins may trigger hypersensitivity responses and immune-mediated injury. Concurrently, oxidative stress generated by reactive oxygen species amplifies epithelial damage and stimulates fibroblast activation, promoting interstitial fibrosis analogous to that observed in other occupational lung diseases, such as byssinosis or silicosis [5]. Despite the biological plausibility and documented symptom burden, systematic pulmonary surveillance remains uncommon in small-scale Indian industries. Existing studies are often geographically restricted, limited by modest sample sizes, and characterized by heterogeneity in methodology, thereby limiting comparability and generalizability of findings.

A critical clinical gap persists in the form of limited standardized spirometric data for Indian rice mill workers assessed under controlled and comparable conditions. Spirometry represents a validated, non-invasive, and reproducible tool for evaluating pulmonary mechanics and detecting early functional impairment [6]. Key parameters, including forced vital capacity (FVC), forced expiratory volume in one second (FEV₁), and peak expiratory flow rate (PEFR), serve as sensitive indicators of declining lung function. The FEV₁/FVC ratio further facilitates differentiation between obstructive and restrictive ventilatory defects, thereby providing insight into the predominant pattern of impairment. However, the absence of adequately controlled, age- and anthropometry-matched comparative data hampers precise characterization of exposure-related respiratory effects within this workforce [7,8].

In light of these considerations, the present study was undertaken to systematically compare spirometric parameters between rice mill workers with chronic occupational exposure and matched unexposed controls. By addressing the paucity of standardized pulmonary function data in this Indian occupational setting, the study aims to generate clinically relevant evidence to inform early detection strategies, workplace risk assessment, and formulation of targeted occupational health policies for this vulnerable population.

## Materials and methods

This cross-sectional comparative study was conducted in the Department of Physiology, Karpaga Vinayaga Institute of Medical Sciences and Research Centre, Madhuranthagam, Chengalpattu, Tamil Nadu, India, from January to June 2025. Ethical approval was obtained from the Institutional Ethics Committee (Reg. No. ECR/1425/Inst/TN/2023). All procedures were conducted in accordance with the Declaration of Helsinki. Written informed consent was obtained from all participants before enrollment.

Participant selection

For a cross-sectional study evaluating differences in mean spirometric parameters between an exposed group (rice mill workers) and a control group, the sample size for comparing two independent means can be calculated using the following formula:

$n = \frac{2\sigma^{2}\left(Z_{\alpha/2} + Z_{\beta}\right)^{2}}{(\mu_{1} - \mu_{2})^{2}}$

Where: n = required sample size per group σ = pooled standard deviation μ1 = mean in the exposed group μ2 = mean in the control group (μ1 − μ2) = expected difference in means Zα/2 = 1.96 (5% significance level) Zβ = 0.84 (80% power) Rearranging the formula: $(\mu_{1} - \mu_{2}) = \sqrt{\frac{2\sigma^{2}\left(Z_{\alpha/2} + Z_{\beta}\right)^{2}}{n}}$ Substituting values: $(\mu_{1} - \mu_{2}) = \sqrt{\frac{2\sigma^{2}(1.96 + 0.84)^{2}}{50}}$ = $\sqrt{\frac{2\sigma^{2}(2.8)^{2}}{50}}$ = $\sqrt{\frac{15.68\sigma^{2}}{50}}$ = $\sqrt{0.3136\sigma^{2}}$ = $0.56\sigma$ Thus, a sample size of 50 participants per group provides 80% power to detect a difference of approximately 0.56 standard deviations between groups [2].

Study Group (n = 50)

Male rice mill workers aged 30-60 years, employed for ≥1 year in the milling process (dehusking, polishing, or packaging sections).

Control Group (n = 50)

Age and BMI-matched males working in administrative or teaching occupations with no exposure to industrial dust.

Inclusion Criteria

The following were included: male participants aged 30-60 years; minimum one year of employment in rice milling (study group); non-smokers; and willingness to undergo spirometry.

Exclusion Criteria

Females, those with a history of smoking or alcohol abuse; known cases of asthma, COPD, and tuberculosis; those with a history of cardiovascular diseases; those with a history of acute respiratory infection within four weeks; those with neuromuscular disorders or thoracic and spine abnormalities; and those with an inability to perform reproducible spirometry were excluded.

Data collection and clinical assessment

Participants underwent a structured interview recording demographic data, duration of work exposure, medical history, and occupational practices. Anthropometric measurements--height (cm), weight (kg), and BMI (kg/m²)--were obtained using standardized equipment. Environmental observations were made regarding dust control systems, use of masks, and ventilation status within the mills [9].

Spirometry procedure

The following parameters were measured: FVC (L) - maximum volume exhaled forcefully after full inspiration; FEV₁ (L) - volume exhaled in the first second; FEV₁/FVC (%) - ratio indicating ventilatory pattern; PEFR (L/s) - peak expiratory flow rate reflecting large airway patency.

Pulmonary function testing was performed using a calibrated Medicaid Smart USB spirometer (Medicaid Systems, Jalandhar, Punjab, India) in accordance with the 2019 standards for spirometry recommended by the American Thoracic Society and European Respiratory Society [10]. The spirometer was calibrated daily as per the manufacturer's instructions to ensure accuracy and reliability. All measurements were conducted between 9:00 a.m. and 12:00 p.m. to minimize diurnal variation in lung function. Participants were instructed to refrain from heavy meals, smoking, caffeine intake, and strenuous physical activity for at least two hours prior to testing. Anthropometric data, including age, sex, height, and weight, were recorded before the procedure to compute predicted spirometric values [11].

Spirometry was performed with the participant in a seated position, using a nose clip to prevent nasal air leakage. After a clear demonstration of the maneuver, each subject was instructed to take a maximal inspiration to total lung capacity, followed immediately by a rapid, forceful, and sustained expiration into a sterile disposable mouthpiece. Expiration was continued for a minimum of six seconds or until no further air could be expelled. Strong verbal encouragement was provided to ensure maximal effort and reproducibility. A minimum of three acceptable forced expiratory maneuvers were obtained from each participant [12]. Acceptability criteria included an adequate start of expiration without hesitation, absence of coughing during the first second, no early termination, and satisfactory exhalation duration. Repeatability was confirmed when the two highest values of FVC and FEV₁ were within 150 mL of each other. If necessary, additional attempts (up to a maximum of eight) were allowed [13].

The highest recorded values of FVC, FEV₁, and PEFR were documented. The FEV₁/FVC ratio was calculated to determine ventilatory patterns. All tests were performed by trained personnel under standardized conditions to maintain quality control.

Data analysis

Data were analyzed using IBM SPSS Statistics version 31 (IBM Corp., Armonk, NY, US). Continuous variables were summarized as mean ± standard deviation. Intergroup comparisons were performed using the independent-samples t-test after assessment of distributional assumptions. A two-sided p-value < 0.05 was considered statistically significant.

## Results

The baseline demographic and anthropometric characteristics (Table 1) of the study and control groups were comparable. The mean age of rice mill workers (42.7 ± 10.6 years) was nearly identical to that of the control group (42.6 ± 5.7 years), with no statistically significant difference (p = 0.95). Similarly, no significant differences were observed in height (p = 0.47), weight (p = 0.92), or body mass index (BMI) (p = 0.35).

**Table 1:** Comparison of baseline characteristics between the study and control groups Continuous variables are presented as mean ± standard deviation (SD) and were compared using the independent-samples t-test. BMI, body mass index

Parameter	Study Group (Mean ± SD)	Control Group (Mean ± SD)	t-value	p-value
Age (years)	42.7 ± 10.6	42.6 ± 5.7	0.05	0.95
Height (cm)	173.1 ± 14.1	174.7 ± 6.8	0.71	0.47
Weight (kg)	78.94 ± 22.6	78.6 ± 11.01	0.08	0.92
BMI (kg/m²)	26.4 ± 3.5	25.7 ± 3.3	0.92	0.35

These findings indicate adequate matching between the two groups with respect to major determinants of pulmonary function such as age and body composition.

Pulmonary function testing (Table 2) revealed marked and statistically highly significant reductions in ventilatory parameters among rice mill workers as compared to controls.

**Table 2:** Comparison of pulmonary function test (PFT) parameters between the study and control groups Data are presented as mean ± standard deviation (SD). Intergroup comparisons were performed using the independent samples t-test, and corresponding t-values are reported. A p-value < 0.05 was considered statistically significant. FVC: forced vital capacity (L); FEV₁: forced expiratory volume in the first second (L); PEFR: peak expiratory flow rate (L/s); FEV₁/FVC ratio: forced expiratory volume in the first second to forced vital capacity ratio (%)

PFT Parameter	Study Group (Mean ± SD)	Control Group (Mean ± SD)	t-value	p-value
FVC (L)	1.96 ± 0.22	3.88 ± 0.98	13.4	<0.05
FEV₁ (L)	1.69 ± 0.21	3.28 ± 0.79	13.6	<0.05
PEFR (L/s)	4.47 ± 1.89	7.83 ± 1.59	9.7	<0.05
FEV₁/FVC (%)	87.97 ± 0.21	81.40 ± 1.57	29.3	<0.05

The mean FVC was substantially lower in the exposed group (1.96 ± 0.22 L) compared with controls (3.88 ± 0.98 L), representing nearly a 50% reduction (p < 0.05).

Similarly, the mean FEV₁ was significantly reduced in the study group (1.69 ± 0.21 L) compared with controls (3.28 ± 0.79 L; p < 0.05).

PEFR, an indicator of large airway flow dynamics and expiratory muscle effort, was also significantly decreased among rice mill workers (4.47 ± 1.89 L/s vs. 7.83 ± 1.59 L/s; p < 0.05).

The FEV₁/FVC ratio was significantly higher in the study group (87.97 ± 0.21%) compared to controls (81.40 ± 1.57%; p < 0.05).

Pulmonary function testing revealed significantly lower FVC, FEV₁, and PEFR among rice mill workers than controls. The FEV₁/FVC ratio was significantly higher in the exposed group. This pattern, characterized by reduced FVC with a preserved or elevated FEV₁/FVC ratio, is suggestive of a restrictive spirometric pattern; however, definitive confirmation of restrictive lung disease would require static lung volume measurement.

## Discussion

The study and control groups were comparable with respect to age and body mass index (BMI), thereby minimizing the influence of these important confounding variables on pulmonary function outcomes. The mean age of the study group (42.7 ± 10.6 years) was nearly identical to that of the control group (42.6 ± 5.7 years), with no statistically significant difference (p = 0.95). Age is a well-recognized determinant of respiratory performance, as advancing age is associated with reduced elastic recoil, diminished chest wall compliance, and gradual declines in FEV₁ and FVC due to structural and physiological changes within the respiratory system. Standardization for age is therefore essential in spirometric comparisons, as emphasized by international spirometry guidelines. Studies that fail to control for age differences may overestimate occupational effects because lung function naturally declines with aging. Similar occupational investigations have also emphasized age-matched comparison groups to reduce demographic bias in pulmonary function analysis [14,15].

Similarly, BMI was comparable between the two groups (26.4 ± 3.5 kg/m² vs. 25.7 ± 3.3 kg/m²; p = 0.35), further strengthening the internal validity of the study. Increased BMI is known to influence respiratory mechanics through reduced lung compliance, decreased functional residual capacity, and limitation of diaphragmatic excursion. The association between elevated BMI and restrictive ventilatory changes has been clearly described by Shanmugasundaram K et al. [16], who demonstrated that obesity contributes to reductions in lung volumes independent of occupational exposure. Although some occupational studies have reported higher BMI among exposed workers contributing to additive restrictive effects, the absence of a significant BMI difference in the present study supports the interpretation that the observed pulmonary impairment is primarily attributable to occupational dust exposure rather than anthropometric variation [17].

Dust pollution remains a major occupational hazard with well-established adverse respiratory effects. In the present study, pulmonary function parameters among rice mill workers exposed to rice husk dust were compared with age- and BMI-matched unexposed controls to evaluate the impact of chronic occupational exposure. The exposed group demonstrated significant reductions in FVC, FEV₁, and PEFR, whereas the FEV₁/FVC ratio was significantly increased, indicating measurable impairment of pulmonary function. These findings reflect the cumulative physiological consequences of repeated inhalation of organic and inorganic particulate matter generated during milling operations.

The milling process itself contributes substantially to airborne dust generation. Approximately 78% of paddy input is recovered as rice, broken rice, and bran, while nearly 22% becomes husk, which is frequently utilized as fuel for steam generation during parboiling. Combustion and handling of husk produce continuous dust emission, creating a persistently contaminated occupational environment. Chronic exposure to such particulate matter provides a plausible explanation for the decline in spirometric indices observed among exposed workers [18].

The reductions in FVC, FEV₁, and PEFR observed in the exposed group are consistent with impaired pulmonary function associated with chronic dust exposure. The pattern of reduced FVC accompanied by a preserved or elevated FEV₁/FVC ratio may suggest a restrictive spirometric pattern, although this cannot be confirmed without lung volume measurements. The reduction in PEFR may also reflect impaired expiratory flow dynamics [19]. Reduction in FVC may indicate structural alterations involving bronchial walls and elastic lung components, producing a predominantly restrictive ventilatory pattern [20,21]. Decline in FEV₁ suggests concomitant early airway obstruction resulting from inflammatory responses to inhaled particulates. Previous investigations among rice mill workers have also demonstrated reductions in peak expiratory flow rate associated with occupational dust exposure [22].

Section-wise analysis revealed significant differences in PEFR among workers employed in different milling areas. This variation may be explained by differential exposure to airborne endotoxins capable of inducing inflammatory responses within the broncho-pulmonary tree [23]. Dust concentrations are typically higher in operational sections characterized by large working spaces and continuous material movement, which facilitate aerosolization and inhalation of particulate matter [24]. Previous literature similarly identifies dumping, feeding, cleaning, dehusking, and polishing sections as major dust hazard zones within rice mills [25]. Rotational deployment of workers across these sections may therefore represent a practical strategy to reduce cumulative exposure.

Public health and clinical relevance

The findings of the present study have important implications for occupational health, particularly in developing countries where rice milling continues to employ a large workforce under conditions of significant dust exposure. Workers involved in rice milling operations are frequently exposed to organic dust generated during activities such as husking, polishing, and grain handling. Prolonged inhalation of these airborne particles has been consistently associated with respiratory symptoms and measurable declines in pulmonary function among exposed workers [26,27].

From a clinical standpoint, early identification of respiratory impairment among exposed workers is essential in preventing the progression to chronic respiratory disease. Periodic spirometric surveillance programs and occupational health screening can facilitate early detection of lung function decline, enabling timely clinical intervention and implementation of preventive measures [28].

Furthermore, engineering interventions aimed at reducing airborne dust concentration, including improved workplace ventilation systems, dust extraction mechanisms, and mechanized handling processes, have demonstrated significant effectiveness in minimizing occupational exposure in rice milling environments [29]. In addition to engineering controls, the consistent use of personal protective equipment, such as respiratory masks, and worker education regarding occupational hazards are essential components of effective preventive strategies.

At a broader level, strengthening occupational safety regulations and implementing comprehensive workplace health policies are necessary to reduce the burden of respiratory morbidity among workers in agricultural processing industries. Given the substantial number of individuals employed in rice milling sectors across low- and middle-income countries, addressing occupational dust exposure through preventive interventions could significantly improve long-term respiratory health outcomes and reduce the public health burden associated with occupational lung diseases [30].

Limitations and future direction

The exclusion of female participants represents a potential source of selection bias. This was primarily due to their limited representation in the study setting and differences in occupational exposure patterns, which could introduce variability in spirometric outcomes. While this approach improved group homogeneity and internal validity, it restricts the generalizability of the findings, as the results cannot be extrapolated to female workers. Future studies should include adequately powered female cohorts to evaluate potential gender-based differences in pulmonary function.

The observed response trend indicates that prolonged exposure may result in progressive and potentially irreversible pulmonary impairment, highlighting the need for early detection through periodic spirometric surveillance and timely intervention. Preventive strategies should include engineering controls (dust extraction systems, wet milling, improved ventilation), administrative measures (worker rotation and scheduled rest breaks), mandatory use of appropriate personal protective equipment, such as N95 respirators, structured health education on respiratory hazards, and strict enforcement of occupational exposure limits.

However, this study has notable limitations. The small sample size (n = 100) may limit statistical power and generalizability. The cross-sectional design precludes causal inference and temporal assessment of lung function decline. Inclusion of only males aged 30-60 years restricts external validity. Environmental dust levels were not directly quantified, limiting objective exposure assessment and dose-response evaluation. Additionally, the absence of post-shift symptom assessment and inflammatory biomarker analysis constrains mechanistic interpretation. Future longitudinal studies incorporating comprehensive environmental monitoring and cytokine profiling are warranted to establish causality and clarify underlying pathophysiological mechanisms.

## Conclusions

The present study demonstrates that rice mill workers had significantly lower spirometric values than unexposed controls. The combination of reduced FVC and FEV₁ with a preserved or elevated FEV₁/FVC ratio suggests a restrictive spirometric pattern, although confirmatory lung volume testing was not performed. These findings support the use of spirometry as a screening tool in occupational surveillance programs and underscore the need for workplace dust-control measures and larger longitudinal studies.

## References

[1] Chaudhuri S, Jaison MK, Chattopadhyay B, Paul KK, Sengupta T (2024). Pattern of occupational lung disease among industrial workers attending a medical college of Eastern India. J Family Med Prim Care.

[2] Biswas M, Pranav PK, Nag PK (2023). Effect on pulmonary functions of dust exposed rice mill workers in comparison to an unexposed population. Work.

[3] Ghosh T, Gangopadhyay S, Das B (2014). Prevalence of respiratory symptoms and disorders among rice mill workers in India. Environ Health Prev Med.

[4] Kumar Chhetry BS, Tada T, Dewangan KN, Kumar P (2024). Evaluation of dust and endotoxin exposure among rice mill workers in northeast India. Toxicon.

[5] Sundaram P, Vinutha Shankar MS, Aswathappa J, Kutty K (2019). Pulmonary function impairment in rice mill workers of a rural area. Indian J Physiol Pharmacol.

[6] Graham BL, Steenbruggen I, Miller MR (2019). Standardization of spirometry 2019 update. An official American Thoracic Society and European Respiratory Society technical statement. Am J Respir Crit Care Med.

[7] Stanojevic S, Kaminsky DA, Miller MR (2022). ERS/ATS technical standard on interpretive strategies for routine lung function tests. Eur Respir J.

[8] Bhakta NR, McGowan A, Ramsey KA (2023). European Respiratory Society/American Thoracic Society technical statement: standardisation of the measurement of lung volumes, 2023 update. Eur Respir J.

[9] Peter JB, Pandiyan I (2025). Evaluation of occupational health hazards among industrial workers in a Irungattukottai village- a cross-sectional study. J Pioneer Med Sci.

[10] Haynes JM (2018). Basic spirometry testing and interpretation for the primary care provider. Can J Respir Ther.

[11] Cengiz SK, Savas IK, Coskun E, Erten HÇ, Cömert SS (2025). Conformity of spirometric tests with acceptability criteria and assessment of confounding factors in routine clinical practice. Ann Thorac Med.

[12] Chittaluru PK, Korra RK, Asuri VK, Annakula P, Gmm R (2021). An analytical cross-sectional study to compare pulmonary function and respiratory morbidity-related quality of life between construction workers with age-and gender-matched controls. Indian J Occup Environ Med.

[13] Saini M, Shah IA, Thasale S, Sivaperumal P, Kulkarni N (2025). Quantifying and modeling respirable dust and crystalline silica exposure in rice mills using the CART algorithm. Sci Rep.

[14] Satapathy AK, Das RR, Mahapatro S, Panigrahi MK, Bandopadhaya D (2022). Effect of body mass index (BMI) on pulmonary functions in children of 6-14 years of age: a cross-sectional study. J Family Med Prim Care.

[15] Elsaidy WH, Alzahrani SA, Boodai SM (2024). Exploring the correlation between body mass index and lung function test parameters: a cross-sectional analytical study. BMC Res Notes.

[16] Shanmugasundaram K, Bade G, Sampath M, Talwar A (2023). Effect of obesity on airway mechanics. Indian J Endocrinol Metab.

[17] Ponce MC, Sankari A, Sharma S (2025). Pulmonary function tests. StatPearls [Internet].

[18] Choudhury SA, Rayhan A, Ahmed S (2023). Frequency of respiratory symptoms among rice mill workers in Bangladesh: a cross-sectional study. Health Sci Rep.

[19] Upadhyay K, Bagepally BS, Balachandar R, Sheth A, Viramgami A (2025). Pulmonary function among flour mill workers: a systematic review and meta-analysis. BMC Public Health.

[20] Farokhi A, Heederik D, Smit LA (2018). Respiratory health effects of exposure to low levels of airborne endotoxin - a systematic review. Environ Health.

[21] Darbakk C, Graff P, Olsen R (2024). Assessment of occupational exposure to inhalable aerosols in an instant powdered food manufacturing plant in Norway. Saf Health Work.

[22] Pranav PK, Biswas M (2016). Mechanical intervention for reducing dust concentration in traditional rice mills. Ind Health.

[23] Ashuro Z, Debela BG, Daba C, Hareru HE, Abaya SW, Byrne AL (2024). The effect of occupational exposure to organic dust on lung function parameters among African industrial workers: a systematic review and meta-analysis. Front Public Health.

[24] Demeke D, Haile DW (2018). Assessment of respiratory symptoms and pulmonary function status among workers of flour mills in Addis Ababa, Ethiopia: comparative cross-sectional study. Pulm Med.

[25] Rana MC, Naskar S, Roy R, Das DK, Das S (2018). Respiratory morbidity among rice mill workers in an urban area of Burdwan district, West Bengal: a cross-sectional study. Indian J Occup Environ Med.

[26] Anlimah F, Gopaldasani V, MacPhail C, Davies B (2023). A systematic review of the effectiveness of dust control measures adopted to reduce workplace exposure. Environ Sci Pollut Res Int.

[27] Shamsollahi HR, Jahanbin B, Rafieian S, Yunesian M (2021). Particulates induced lung inflammation and its consequences in the development of restrictive and obstructive lung diseases: a systematic review. Environ Sci Pollut Res Int.

[28] He W, Jin N, Deng H (2022). Workers’ occupational dust exposure and pulmonary function assessment: cross-sectional study in China. Int J Environ Res Public Health.

[29] Ayodele CO, Yekinni OS, Olaniyi EO, Udokporo CF (2025). Occupational exposure patterns and respiratory disease manifestations in industrial workers across multiple manufacturing sectors. World J Adv Res Rev.

[30] Cullinan P, Muñoz X, Suojalehto H (2017). Occupational lung diseases: from old and novel exposures to effective preventive strategies. Lancet Respir Med.

